# Potential of peptide‐engineered exosomes with overexpressed miR‐92b‐3p in anti‐angiogenic therapy of ovarian cancer

**DOI:** 10.1002/ctm2.425

**Published:** 2021-05-18

**Authors:** Jiaying Wang, Conghui Wang, Yang Li, Mingyue Li, Tingjia Zhu, Zhangjin Shen, Hui Wang, Weiguo Lv, Xinyu Wang, Xiaodong Cheng, Xing Xie

**Affiliations:** ^1^ Women's Reproductive Health Laboratory of Zhejiang Province Women's Hospital School of Medicine Zhejiang University Hangzhou Zhejiang China; ^2^ Department of Gynecologic Oncology Women's Hospital School of Medicine Zhejiang University Hangzhou Zhejiang China

**Keywords:** anti‐angiogenesis, Apatinib, engineered exosomes, miR‐92b‐3p, ovarian cancer

## Abstract

**Introduction:**

Exosomal microRNA (miRNA) as a mediator of intercellular communication plays an essential part in tumor‐relevant angiogenesis. Therapy against angiogenesis has been demonstrated to have a remarkable antitumor efficacy in various malignancies, but not as expected in ovarian cancer.

**Methods:**

Exosomes were isolated by ultracentrifugation. Exosomal miRNA sequencing and gene function experiments were used to identify the differential expressed miRNAs in exosomes and their mRNA targets. SKOV3 cell line that stably overexpressed miR‐92b‐3p was constructed by lentivirus. In vitro, angiogenesis was analyzed by tube formation assay and migration assay. The angiogenic and antitumor effects in vivo were assessed in zebrafish and nude mouse models. Combination index was calculated to assess the synergetic inhibition of angiogenesis between miR‐92b‐3p and Apatinib. Peptides were conjugated with exosomal membranes to obtain engineered exosomes.

**Results:**

Ovarian cancer cell‐derived exosomes facilitated the angiogenesis and migration capability of vascular endothelial cells in vitro and in vivo. The expression of miR‐92b‐3p was much lower in ovarian cancer cell‐derived exosomes than that in immortalized ovarian epithelial cell‐derived exosomes. The exosomal miR‐92b‐3p modulated tumor‐associated angiogenesis via targeting SOX4. Besides, Peptide‐engineered exosomes with overexpressed miR‐92b‐3p showed the stronger abilities of anti‐angiogenesis and antitumor than parental exosomes, whether alone or combined with Apatinib.

**Conclusions:**

Our findings demonstrate the effect and mechanism of exosomal miR‐92b‐3p from ovarian cancer cells on tumor‐associated angiogenesis and the potential of artificially generated exosomes with overexpressed miR‐92b‐3p to be used as anti‐angiogenic agent, which may provide a new approach for anti‐angiogenic therapy of ovarian cancer.

## Graphical abstract

1. miR‐92b‐3p is significantly lower in exosomes derived from ovarian cancer cells than that derived from normal ovarian epithelial cells.

2. Exosomes with overexpressed miR‐92b‐3p can inhibit the angiogenesis of ovarian cancer.

3. DSPE‐PEG2K‐RGD modified engineered exosomes loaded with miR‐92b‐3p can produce a combined antitumor and anti‐angiogenic effect with Apatinib in nude mice abdominal tumor models.

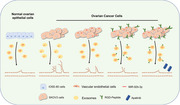

**Abbreviations**
dpf: days post fertilization
dpi: days post injection
DRB: 5,6‐dichlorobenzimidazole 1‐β‐D‐ribofuranoside
EOC: epithelial ovarian cancer
FBS: fetal bovine serum
HUVEC: human umbilical vein endothelial cell line
IHC: immunohistochemistry
miRNA: microRNA
mut: mutant
MVD: micro‐vessels density
TME: tumor microenvironment
VEGFR2: vascular endothelial growth factor receptor 2
wt: wild type

## 1 INTRODUCTION

The tumor microenvironment (TME) is strongly linked to the growth and development of tumors.^1^, ^2^ Tumor‐associated angiogenesis has been recognized as an essential event in TME that can be controlled by cancer cells.^3^ In the last decade, anti‐angiogenic agents including antibodies and small molecules showed promise as therapeutics in solid tumors.^4^ The accumulating evidence demonstrates the survival benefit of cancer patients from anti‐angiogenic therapy, such as metastatic colorectal cancer, hepatocellular carcinoma, and others.^5^, ^6^

Epithelial ovarian cancer (EOC) ranks the first fatal disease in female reproductive malignancies.^7^ Despite the advances in chemotherapy and surgery, the 5‐year survival rate of advanced EOC patients is always less than 30% for several decades, due to drug resistance.^8^ Angiogenesis also occurs in ovarian cancer and is closely related to poorer prognosis of the patients,^9^, ^10^, ^11^, ^12^ but anti‐angiogenic therapy for ovarian cancer is not as effective as for other solid tumors. For instance, Apatinib, a novel tyrosine kinase inhibitor (TKI) of vascular endothelial growth factor receptor 2 (VEGFR2) with a high specificity, has been clinically proved effective in various solid tumors.^13^, ^14^ However, the evidence of benefits from Apatinib for ovarian cancer is limited, mainly from recurrent cases.^15^, ^16^ So far, there is lack of data on the use of TKIs, including Apatinib, in the first‐line chemotherapy in ovarian cancer. Finding the new targeting angiogenesis approach, alone or combined with current anti‐angiogenic agents, would help to improve ovarian cancer patients’ prognosis.

Exosomes, as a vesicle smaller than 150 nm in diameter, are enrich in microRNAs (miRNAs), proteins, lipids, and others^17^ and act as mediator of intercellular communication.^18^, ^19^, ^20^ miRNA is a kind of noncoding RNA molecule and typically comprises of approximately 20 nucleotides. Recent studies have reported the role of exosomal miRNAs in promoting tumor angiogenesis.^21^, ^22^, ^23^ With the advance of exosome studies, the exosomal miRNA‐based therapeutics in cancer is constantly developing, but the potential of exosomal miRNAs, especially miRNAs packed in engineered exosomes, as anti‐angiogenic agents in cancers is still completely unclear up to date.

Here, we identified that lower exosomal miR‐92b‐3p expression was associated with ovarian cancer angiogenesis, and found that overexpressed exosomal miR‐92b‐3p suppressed tumor‐related angiogenesis in vitro and in vivo, and exosomal miR‐92b‐3p and Apatinib had a strong synergic effect on anti‐angiogenesis in vitro. We further generate peptide‐engineered exosomes with miR‐92b‐3p overexpression and validated that the engineered exosomes alone and combined with Apatinib inhibited the tumor growth and angiogenesis in vivo. Our findings may provide a new approach for ovarian cancer anti‐angiogenic therapy.

## 2 METHODS

### 2.1 Cell culture

Human ovarian cancer cell lines SKOV3 and A2780 were obtained as described previously.^24^ Human ovarian epithelial cell line IOSE‐80 was kindly provided by Prof. Lu Yan, Zhejiang University. Human umbilical vein endothelial cell line (HUVEC) was purchased from Shanghai Zhongqiao Xinzhou Biotechnology (#8000, Sciencell). 293T cell line was purchased from ATCC (CRL‐3216, ATCC). SKOV3 or A2780 cells were cultured in McCoy's 5A or RPMI 1640 (L630KJ, L210KJ, Basal Media), IOSE‐80 and 293T cells were cultured in DMEM (L110KJ, Basal Media). Medium mentioned above was added with 10% fetal bovine serum (FBS) (11011‐8611, Everyday Green). Endothelial cell medium (#1001, Sciencell) was used for HUVECs’ culturing.

### 2.2 Exosome isolation, quantitation, and characterization

The isolation of exosomes was performed according to the protocols descripted before.^25^ An Optima XPN‐100 ultracentrifuge (Beckman) with a Type 70Ti rotor was used for the ultracentrifugation (all steps were performed at 4°C). After resuspended in PBS, the exosomes were filtered by 0.22‐μm filters (SLGP033RB, Millipore). Exosomes were quantized by BCA Protein Assay Kit (P0012, Beyotime). Exosomes were prepared, fixed as described previously,^26^ and observed by an electron microscopy (Tecnai G2 Spirit) at an acceleration voltage of 80 kV. CD63 and Hsp70 were used as positive markers for exosomes.

### 2.3 Exosomes for cell treatment and uptake assay

HUVECs plated in six‐well plates (2 × 10^5^ cells per well) were fed with different exosomes at 50 μg/ml for 48 h. The different exosomes were labeled by PKH67 (PKH67GL‐1KT, Sigma‐Aldrich) and incubated with HUVECs for 24 h retrospectively. After incubation, HUVECs were observed under confocal microscopy (FV10i, Olympus). TRITC Phalloidin (40734ES75, Yeasen) was used to stain the actin cytoskeleton, and DAPI (ab104139, Abcam) was used for nucleus staining.

### 2.4 Western blotting

The cells or exosomes were lysed with RIPA (R0010, Solarbio), and protein samples with equivalent amount were separated by SurePAGE gels (M00665, GenScript) and transferred onto 0.2‐μm PVDF membranes (1620177, Bio‐Rad) by the eBlot L1 protein transfer system (L00686C, GenScript). At RT, 5% milk was used to block the membranes. Primary antibodies and secondary antibodies were used according to the protocols after blocking. FDbio‐Pico ECL kit (FD8000, Fudebio‐tech) was used for showing the blots. The antibodies referred are given in Table S1.

### 2.5 Migration and tube formation assays

After the pretreatments of HUVECs with various exosomes for 48 h in six‐well plates, the tube formation and migration ability were tested. The 24‐well transwells (8.0 μm, Falcon) were used for assessing migration ability of HUVECs. Pretreated HUVECs were harvested and 2 × 10^4^ cells resuspended in 200 μl ECM without FBS were plated in the upper chamber of a transwell unit, while the lower chamber was added with 500 μl ECM with 10% exosome‐free FBS. After 4 or 6 h, cells on the lower surface of the chamber were fixed with 4% paraformaldehyde, stained with 0.5% crystal violet and counted. The μ‐Slide Angiogenesis (81506, ibidi) was used for the tube formation assay. HUVECs (1 × 10^4^ cells) in ECM with 2% FBS were seeded on the μ‐Slide that was prechilled and coated with 10 μl of Matrigel (356234, BD). After 3 h, the total tubes were quantified by image analysis of ImageJ.

### 2.6 RNA sequencing, extraction, and qRT‐PCR analysis

Retrospectively, 50 ml of supernatant of each cultured cell line was collected for exosomes extraction and exosomal miRNA sequencing on an Illumina Hiseq 2500 platform by RiboBio Co., Ltd. Total RNA was isolated by Trizol following the instructions. PrimeScript RT reagent kit (RR047A, Takara) and TB Green Premix Ex Taq (RR420A, Takara) were used for the RNA reverse transcription and PCR analysis. All mRNA and exosomal miRNA quantification was done separately using GAPDH and U6 as internal reference. Sequences of primers referred are listed in Table S2.

Criteria for selecting candidate miRNA were as follows: miRNAs with (a) significantly different expression in both IOSE‐80/exo versus SKOV3/exo and IOSE‐80/exo versus A2780/exo sequencing; (b) |log_2_(fold change)| > 1.5 and raw data values >100; (c) the agreement between the results of the qRT‐PCR and exosomal miRNA sequencing; (d) function associated with angiogenesis.

### 2.7 Lentiviruses, plasmids, siRNA, and miRNA transfection

Transient transfections of negative controls, miR‐92b‐3p mimics, and inhibitor were performed by FECT according to the protocols. The reagents were all purchased from Ribobio Co. miR‐92b‐3p overexpression lentiviruses and lentiviral construct expressing luciferase were purchased from Genechem and used in the experiments. For inhibition, siRNAs against SOX4 were synthesized by Genepharma and transfected by DharmaFECT transfection reagents (T‐2001‐03, Thermo). The SOX4 overexpression plasmids were constructed with the GV141 vector by GeneChem and transfected into HUVECs using X‐tremeGENE HP DNA transfection reagent (43940400, Roche). Exosomes were transfected with miR‐92b‐3p inhibitor using Exo‐Fect Reagent (EXFT10A‐1, SBI).

### 2.8 Dual luciferase reporter assay

The 3′‐UTRs of SOX4 including wild‐type (wt) or mutant (mut) miR‐92b‐3p binding sites were cloned downstream of the firefly luciferase gene in pmirGLO vector (Vigene). For dual luciferase reporter assay, 293T cells (1 × 10^4^ cells per well) cotransfected with miRNA mimics and pGLO‐reporter vectors were seeded in a 96‐well plate. After being transfected for 24 h, the supernatant of lysed cells was used for reporter assay according to the manufacturer's protocols (RG027, Beyotime).

### 2.9 CCK‐8 assay

For cell viability assay, IOSE‐80, SKOV3, and A2780 cells were plated in 96‐well plates (5 × 10^3^ cells/well) and fed with different exosomes at 50 μg/ml for 48 h once cells were adherent to the plate. The CCK‐8 assay was performed using CCK‐8 kit following the manufacturer's protocols (FD3788‐500T, Fudebio‐tech).

### 2.10 Zebrafish models

Breeding Tg (*fli*‐1: EGFP) zebrafish were applied in our experiments and maintained in Zhejiang University zebrafish facility. Embryos were raised and maintained according to standard laboratory protocols. The fish were kindly provided by Prof. Xu Pengfei, Zhejiang University. 0.1 ng dre‐miR‐92b‐3p mimics (Ribobio Co.) were injected into one to four‐cell‐stage embryos by a microinjector (WPI SYS‐PV830). Fluorescence was observed on individual embryo 2 days post injection (dpi) by a stereomicroscope. To facilitate visualization post injection, exosomes were prelabeled with PKH‐26 (PKH26GL‐1KT, Sigma‐Aldrich) according to the protocols. One day post‐fertilization (dpf) embryos were anesthetized in a solution containing 0.003% tricaine (A5040, Sigma) for 10 min. Various exosomes were injected into the yolk sac of each embryo equally. For individual experiment, approximately 20 embryos were injected with the same exosomes. Embryos were treated with Apatinib (1 μM, AiTan) or control DMSO at 1 dpf, and fluorescence of individual embryo was observed at 2 dpi.

### 2.11 Nude mouse models

All animal experiments were approved by Animal Ethical and Welfare Committee of Zhejiang Chinese Medical University, Hangzhou, China (grant number: IACUC‐20180604‐06). Fifty Balb/c nude mice (4–6 weeks, female) were obtained from Silaike Experiment Animal Co., Ltd (China) and injected intraperitoneally with 2 × 10^6^ SKOV3‐luc cells in PBS on day 0. On day 3, mice were randomized into 10 groups for different treatments: PBS, IOSE‐80/exo, SKOV3/exo, SKOV3‐92b/exo, RGD‐SKOV3‐92b/exo, RGD‐SKOV3‐92b/exo transfected with miR‐92b‐3p inhibitor, SKOV3/exo plus Apatinib, SKOV3‐92b/exo plus Apatinib, RGD‐SKOV3‐92b/exo plus Apatinib, RGD‐SKOV3‐92b/exo transfected with miR‐92b‐3p inhibitor plus Apatinib. All groups had a similar starting mean bioluminescence intensity. From day 3, each mouse was intravenously injected with 100 μg exosomes every 3 days for 21 days. Apatinib was administered at 100 mg/kg via oral route daily for 21 days. The bioluminescence intensity was measured by the in vivo imaging system (IVIS) after the intraperitoneal injection of D‐luciferin (150 mg/kg, 40901ES01, Yeasen). The mice were sacrificed under anesthesia on day 24, the intraperitoneal tumors and organs were excised and fixed in 4% paraformaldehyde. Immunohistochemistry (IHC) assay was performed for CD31 staining.

### 2.12 IHC and MVD analysis

IHC analysis was performed as previously described.^27^ The number of blood vessels staining positive for CD31 (11265‐1‐AP, Proteintech) were counted. Micro‐vessels density (MVD) evaluation performed in accordance with following principles. Sections of tumor tissue were screened at 40× first to identify the “hotspot” areas, and the micro‐vessels were counted at 200× in random five fields of the “hotspot.” The number of micro‐vessels in each field is MVD.

### 2.13 Preparation of peptide‐engineered exosomes

SKOV3‐92b cells were cultured in McCoy's 5A medium with DSPE‐PEG2K‐RGD (50 μg/ml) and 10% exosome‐free FBS for 48 h, then RGD‐SKOV3‐92b/exos were isolated according to the former protocols.^28^

### 2.14 Reagents

RGD‐peptide (DSPE‐PEG2K‐RGD) was synthesized by Ruixibio, Xi'an. Apatinib (AiTan) was kindly provided by Hengrui Medicine, Jiangsu, China. 5,6‐Dichlorobenzimidazole 1‐β‐D‐ribofuranoside (DRB) (D1916‐10MG) was purchased from Sigma‐Aldrich. Triton X‐100 (X100‐500 ml) was purchased from Sigma‐Aldrich. RNase A solution (R1030) was purchased from Solarbio.

### 2.15 Statistics

SPSS 23.0 software was used for statistical analyses. Either an unpaired *t*‐test or nonparametric test was used for comparison between two groups. *p*‐Value < .05 was considered indicative of significance.

## 3 RESULTS

### 3.1 Cancer cell‐derived exosomes promote angiogenesis and migration viability of HUVECs in vitro and in vivo

**FIGURE 1:**
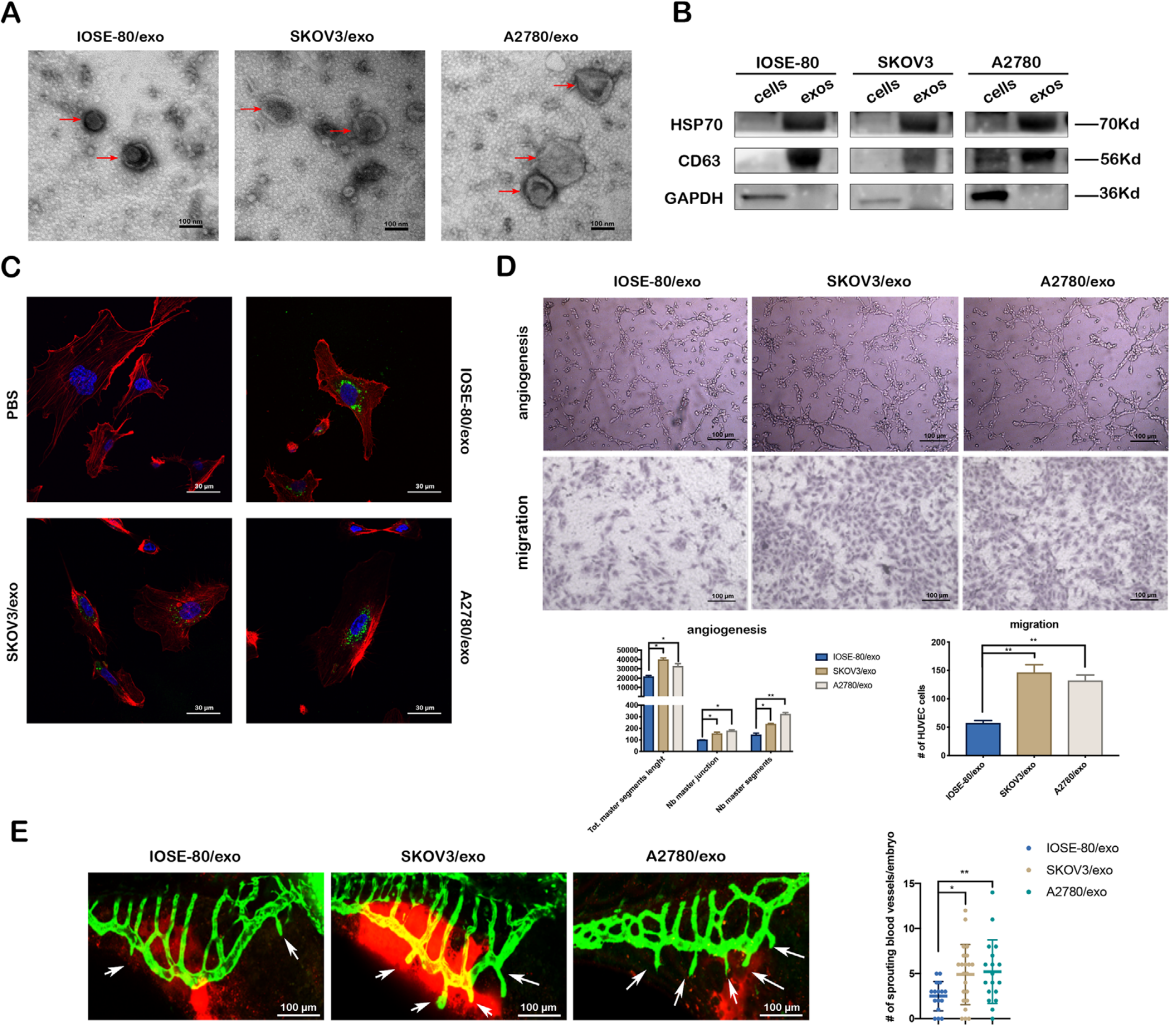
Cancer cell‐derived exosomes promote the angiogenesis and migration viability of HUVECs in vitro and in vivo. (A) Representative TEM images of exosomes (red arrows) (scale bar = 100 nm). (B) Western blotting of the whole‐cell lysates or exosome lysates for the classical exosomal protein markers (CD63 and Hsp70) and GAPDH. (C) Representative confocal microscope images of F‐actin (red), nucleus (blue), and PKH67‐labeled exosomes (green) in HUVECs co‐cultured with various exosomes or PBS for 24 h (scale bar = 30 μm). (D) Representative images of tube formation and migration of HUVECs treated by IOSE‐80/exo, SKOV3/exo, and A2780/exo, respectively (scale bar = 100 μm). Total master segments length, number of master junctions, master nodes, and migration cells were regarded as indicators of angiogenic ability in vitro and assessed by ImageJ (mean ± SD, *n* = 3). (E) Left: Representative confocal images of zebrafish vessel models injected with IOSE‐80/exo, SKOV3/exo, or A2780/exo. The subintestinal vessels (SIV) (green) and exosomes (red) are shown. White arrows indicate newly formed blood vessels. Right: Quantification of the number of ectopic sprouts observed per fish (mean ± SD) injected with IOSE‐80/exo (*n* = 16), SKOV3/exo (*n* = 22), and A2780/exo (*n* = 18), respectively. Data are shown by at least three independent experiments, and the Student's *t*‐test was used to compare differences. *p < .05, **p < .01

Purified exosomes, obtained from supernatant of the immortalized ovarian epithelial cell line (IOSE‐80) and ovarian cancer cell lines (SKOV3 and A2780), showed nanovesicles with typical round or cup shape appearance and an average size around 30–150 nm of diameter under transmission electron microscopy (Figure 1A), and expressed positive exosomal markers as CD63 and HSP70 (Figure 1B). When HUVECs were incubated with above exosomes labeled by PKH‐67 for 24 h, PKH‐67 green fluorescence was seen in HUVECs cultured with exosomes, but not with PBS (Figure 1C). Compared with exosomes secreted by IOSE‐80 cells (IOSE‐80/exo), exosomes secreted by SKOV3 and A2780 cells (SKOV3/exo and A2780/exo, respectively) significantly enhanced the abilities of migration and tube formation of HUVECs at the concentration of 50 μg/ml (Figure 1D). Further, the yolk sac of zebrafish embryos at 24 hpf were injected with IOSE‐80/exo, SKOV3/exo, and A2780/exo, respectively, and fluorescence microscopy showed significantly more new intestinal veins (ISVs) in fish with both SKOV3/exo and A2780/exo compared to those with IOSE‐80/exo after 48 h of injection (Figure 1E).

### 3.2 miR‐92b‐3p acts as a suppressor of tumor‐associated angiogenesis

**FIGURE 2:**
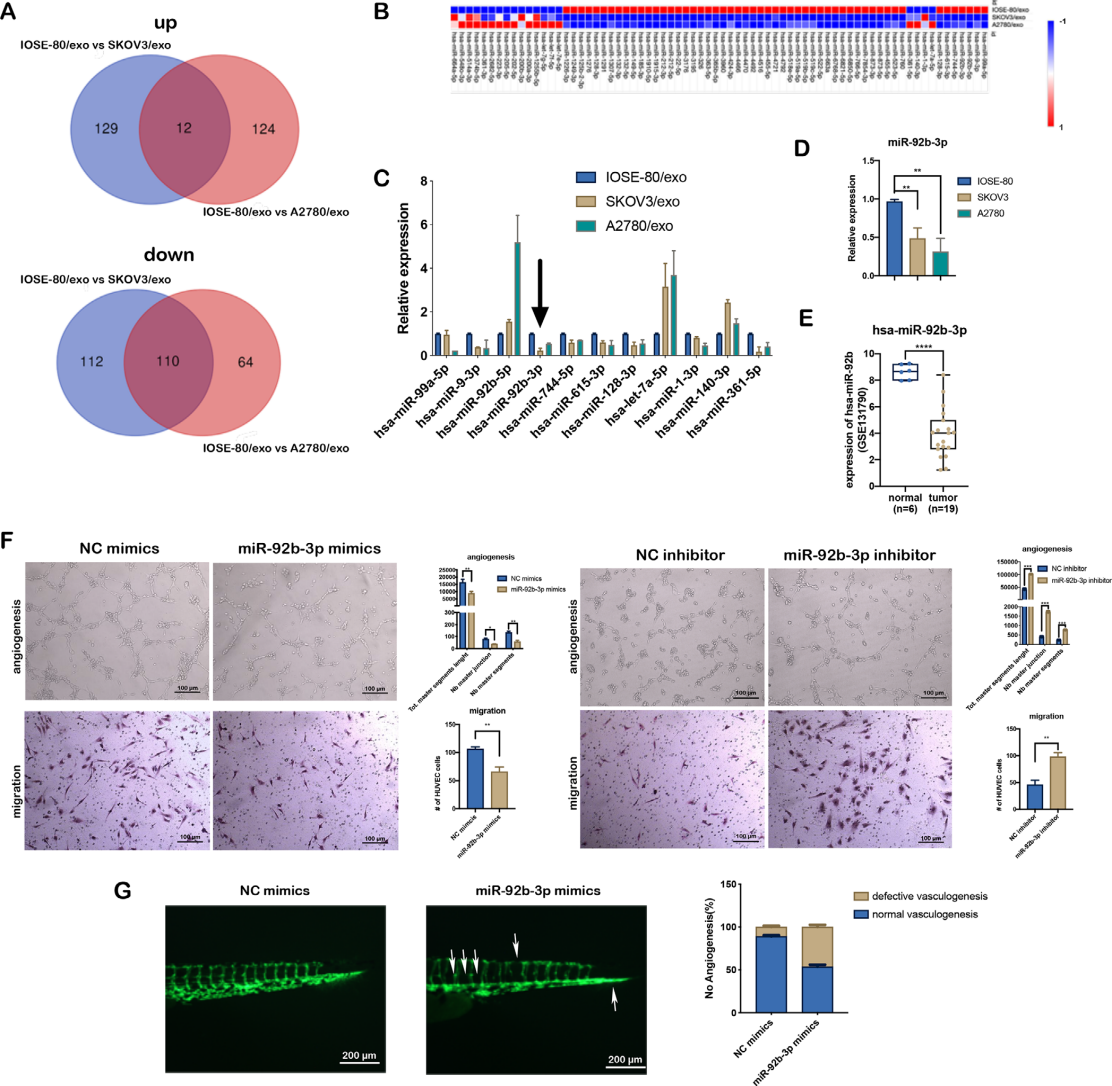
miR‐92b‐3p acts as a suppressor of tumor‐associated angiogenesis. (A) Venn diagram of the overlapping differentially expressed miRNAs (*p* < .05) common to two miRNA sequencings (IOSE‐80/exo vs. SKOV3/exo and IOSE‐80/exo vs. A2780/exo). (B) The heatmap of differentially expressed miRNAs (*p* < .05) (IOSE‐80/exo vs. SKOV3/exo and IOSE‐80/exo vs. A2780/exo). (C) qRT‐PCR validation of difference in miRNA expressions between IOSE‐80/exo and two ovarian cancer cell exosomes (mean ± SD, *n* = 3), data were normalized to the level of U6. Black arrow indicates miR‐92b‐3p. (D) qRT‐PCR analysis on miR‐92b‐3p expression in ovarian cancer cell lines compared to IOSE‐80 cell line (mean ± SD, *n* = 3), data were normalized to the level of U6. (E) Expression of miR‐92b‐3p in ovarian cancer and normal tissue samples in GSE131790 data. (F) Representative images of tube formation and migration of HUVECs transfected with miR‐92b‐3p mimics or miR‐92b‐3p inhibitor (scale bar = 100 μm), compared to the negative controls. Total master segments length, number of master junctions, master nodes, and migration cells were regarded as indicators of angiogenic ability in vitro and assessed by ImageJ (mean ± SD, *n* = 3). (G) Left: Representative images of trunk vasculature in *Tg*(*fli‐1:EGFP*) embryos injected with NC mimics or miR‐92b‐3p mimics (scale bar = 200 μm). Right: Percentage of defective vasculogenesis in zebrafish with NC mimics (*n* = 18) or miR‐92b‐3p mimics (*n* = 23). Data are shown by at least three independent experiments, and the Student's *t*‐test was used to compare differences. *p < .05, **p < .01, ***p < .001, ****p < .0001

Exosomes from donor cells function in recipient cells through cargos they carry, including RNAs and proteins. To explore the effect of exosomal miRNAs on endothelial cells, we performed miRNA comprehensive profiling in exosomes derived from conditional medium of IOSE‐80, SKOV3, and A2780 cells, respectively, by high‐throughput analysis, and found 141 upregulated and 222 downregulated miRNAs between IOSE‐80/exo and SKOV3/exo, 136 upregulated and 174 downregulated miRNAs between IOSE‐80/exo and A2780/exo, of those, 12 upregulated and 110 downregulated miRNAs were overlapped (significant at .05 level of the univariate test) (Figure 2A,B). We selected 11 miRNAs for validation, and found that the expression of eight miRNAs (miR‐99a‐5p, miR‐9‐3p, miR‐92b‐3p, miR‐744‐5p, miR‐615‐3p, miR‐128‐3p, miR‐7a‐5p, and miR‐140‐3p) was consistent with that in miRNA profiling. We then focused on miRNAs that were potentially related to tumor angiogenesis. Of those, miR‐92b‐3p was a miRNA with significant difference and minimum standard deviation between cancer cell and IOSE‐80 exosomes (Figure 2C). Then in IOSE‐80, SKOV3, and A2780 cells, we tested the expressions of miR‐92b‐3p. The results showed that in SKOV3 and A2780 cells, miR‐92b‐3p was significantly lower than that in IOSE‐80 cells (Figure 2D), which were accordant to the expression levels of miR‐92b‐3p in matched exosomes. To further investigate miR‐92b‐3p's role in ovarian cancer tissue samples, we summarized miR‐92b expressions in the GSE131790 data from Gene Expression Omnibus (GEO) database. The miR‐92b expression was lower in tumors (*n* = 19) than normal samples (*n* = 6) (Figure 2E). To assess the potential role of miR‐92b‐3p in angiogenesis and migration of HUVECs, miR‐92b‐3p mimics or inhibitor was transfected, respectively (Figure S1). HUVECs with miR‐92b‐3p overexpression showed significantly weakened abilities of migration and tube formation; however, HUVECs with miR‐92b‐3p inhibition presented significantly enhanced abilities of tube formation and migration (Figure 2F). Further, we injected miR‐92b‐3p mimics into yolk sac of zebrafish embryos at one cell stage. Fluorescence microscope showed remarkably decreased sprouting and vascular endothelial cell migration (white arrowheads) in 48 hpf zebrafish with miR‐92b‐3p mimics injection compared to those with NC mimics (Figure 2G). Our results suggest that miR‐92b‐3p acts as a suppressor of tumor‐associated angiogenesis.

### 3.3 Exosomal miR‐92b‐3p modulates tumor‐associated angiogenesis

**FIGURE 3:**
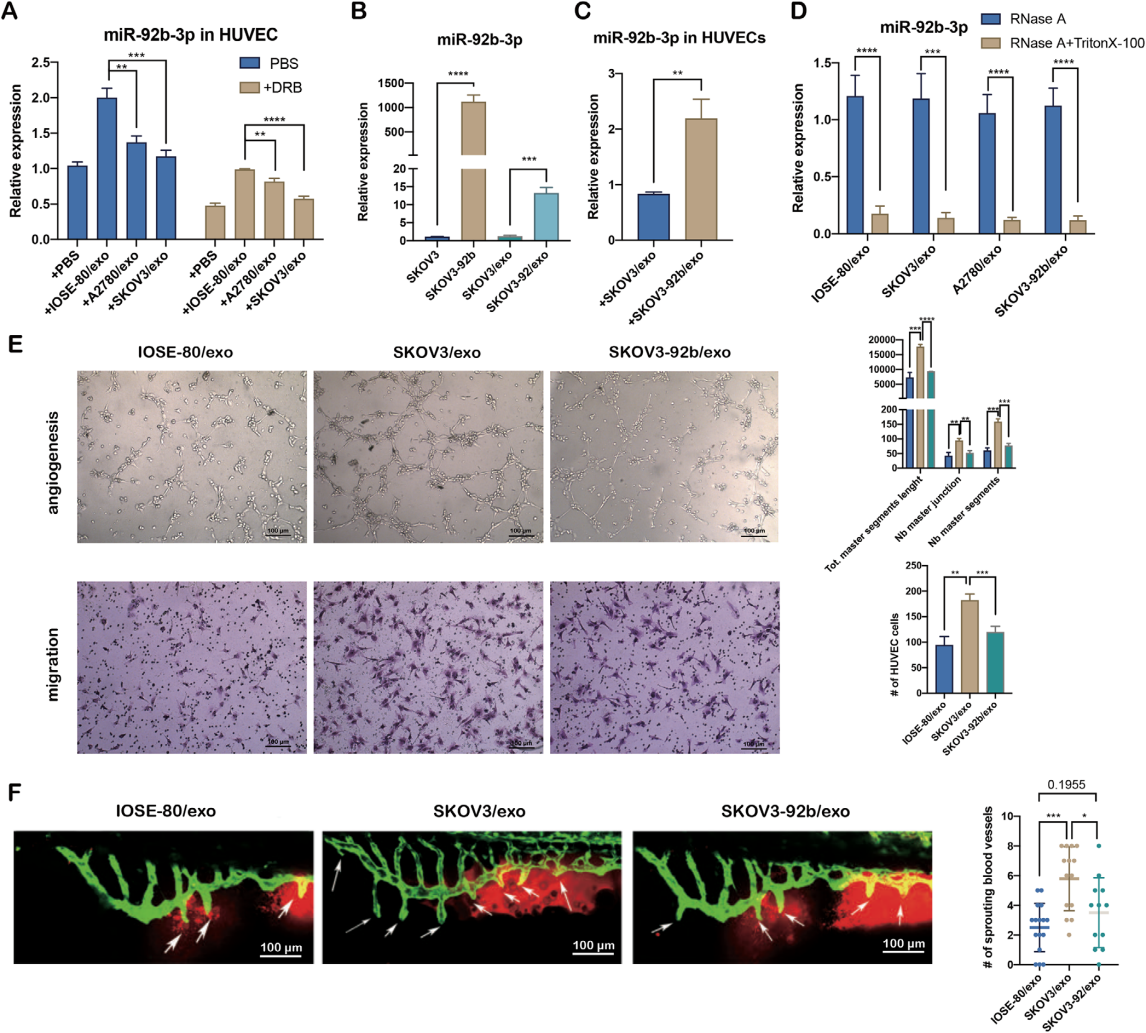
Exosomal miR‐92b‐3p modulates tumor‐associated angiogenesis. (A) qRT‐PCR analysis on the relative expression of miR‐92b‐3p in HUVECs treated with IOSE‐80/exo, SKOV3/exo, and A2780/exo, respectively, for 48 h with or without 5,6‐dichlorobenzimidazole 1‐β‐D‐ribofuranoside (DRB) (20 μm/ml), compared to HUVECs treated with PBS (mean ± SD, *n* = 3). (B) qRT‐PCR analysis on the relative expressions of miR‐92b‐3p in SKOV3 cells, SKOV3‐92b cells, SKOV3/exo, and SKOV3‐92b/exo (mean ± SD, *n* = 3). (C) qRT‐PCR analysis on relative expression of miR‐92b‐3p in HUVECs treated with SKOV3/exo or SKOV3‐92b/exo (mean ± SD, *n* = 3). (D) qRT‐PCR analysis on the expression of miR‐92b‐3p in exosomes after enzyme digestion, with or without destroyed membranes by Triton X‐100 (mean ± SD, *n* = 3). (E) Representative images of tube formation and migration of HUVECs treated with SKOV3/exo and SKOV3‐92b/exo, respectively (scale bar = 100 μm). Total master segments length, number of master junctions, number of master nodes, and migration cell numbers were regarded as indicators of angiogenic ability in vitro and assessed by ImageJ (mean ± SD, *n* = 3). (F) Left: Representative confocal images of the trunk vasculature of zebrafish injected with SKOV3‐NC/exo or SKOV3‐92b/exo (scale bar = 100 μm). Right: Quantification of the number of ectopic sprouts observed per fish (mean ± SD) injected with SKOV3/exo (*n* = 14) or SKOV3‐92b/exo (*n* = 12), respectively. Data are shown by at least three independent experiments and the Student's *t*‐test was used to compare differences. *p < .05, **p < .01, ***p < .001, ****p < .0001

To verify the effect of exosomes on angiogenesis by delivering miR‐92b‐3p from cancer cells to HUVECs, we tested miR‐92b‐3p expressions in HUVECs that were incubated by different exosomes. QRT‐PCR analysis revealed that HUVECs treated with IOSE‐80/exo contained higher level of miR‐92b‐3p than those treated with SKOV3/exo or A2780/exo, meanwhile, miR‐92b‐3p were obviously higher in all above‐mentioned groups compared to the HUVECs treated with PBS (Figure 3A, left). Those phenomena still occurred after RNA synthesis of HUVECs was blocked by DRB, a kind of RNA transcription inhibitor (Figure 3A, right). Considering the significantly lower expression of miR‐92b‐3p in SKOV3/exo than that in A2780/exo and the stronger angiogenic effect of SKOV3/exo than that of A2780/exo, we further constructed a SKOV3 cell line with stable miR‐92b‐3p overexpression (SKOV3‐92b cell line). This cell line expressed 1000‐fold miR‐92b‐3p in cells and 11‐fold miR‐92b‐3p in exosomes (SKOV3‐92b/exo) (Figure 3B). QRT‐PCR showed that SKOV3‐92b/exo delivered more miR‐92b‐3p to HUVECs than SKOV3/exo (Figure 3C). To make sure that miR‐92b‐3p was wrapped in exosomes, membranes of exosomes were destroyed by Triton X‐100 and subjected to digestion with RNase A. The results showed that miR‐92b‐3p was significantly decreasing in multiple exosomes with synergistic treatments of Triton X‐100 and RNase A, compared to those only treated with RNase A (Figure 3D).

Next, tube formation assay and migration assay demonstrated that angiogenesis and migration of HUVECs treated with SKOV3‐92b/exo were inhibited, compared to those with SKOV3/exo (Figure 3E). Zebrafish embryos injected with SKOV3‐92b/exo also presented less newly formed blood vessels compared to those with SKOV3/exo, and similar to IOSE‐80/exo, suggesting that ovarian cancer cells promote the angiogenesis via exosomes delivering less miR‐92b‐3p (Figure 3F).

### 3.4 SOX4 is a target of exosomal miR‐92b‐3p and participates in exosomal miR‐92b‐3p modulating angiogenesis

**FIGURE 4:**
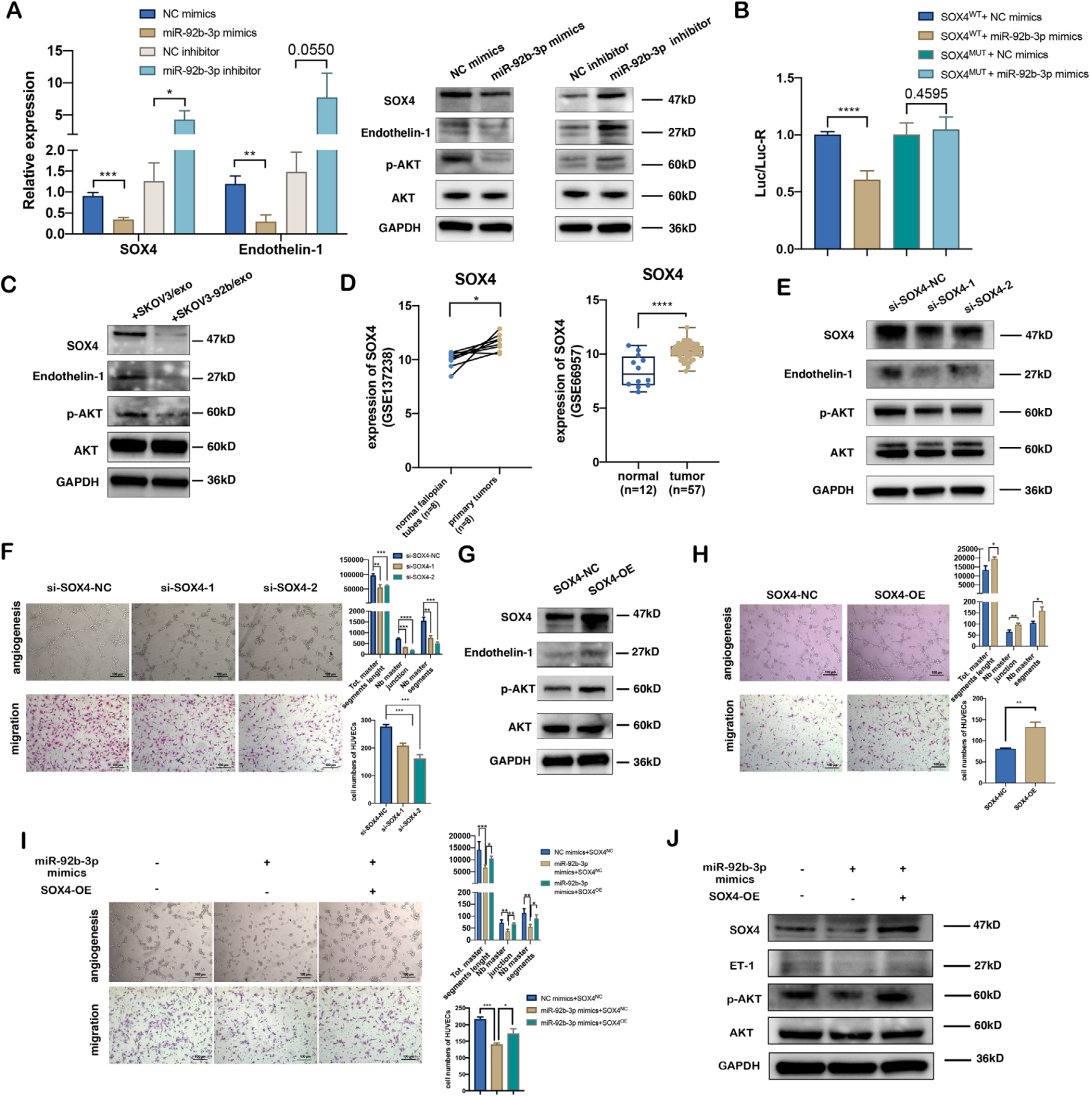
SOX4 is a target of exosomal miR‐92b‐3p and participates in exosomal miR‐92b‐3p modulating angiogenesis. (A) qRT‐PCR analysis (left) and Western blotting (right) of the relative expressions of SOX4, endothelin‐1, and p‐AKT in HUVECs transfected with miR‐92b‐3p mimics or miR‐92b‐3p inhibitor (mean ± SD, *n* = 3). (B) Dual luciferase reporter of verifying the combination between 3′UTR of SOX4 gene and miR‐92b‐3p. The pmirGLO reporters containing 3′UTR of human SOX4 gene with wild‐type (wt) or mutated (mut) miR‐92b‐3p binding sites were used to transfect HUVECs while treating with miR‐92b‐3p mimics or NC mimics (control). Luciferase activity was analyzed at 48 h post transfection (*n* = 6 extracts) and the ratio between Renilla luciferase and firefly luciferase activities (Rluc/Fluc) is shown. (C) Western blotting of the expression level of SOX4, endothelin‐1, and p‐AKT in HUVECs treated with SKOV3/exo and SKOV3‐92b/exo, respectively. (D) Relative expression of SOX4 in ovarian tumors compared to the epithelium of the normal fallopian tubes (GSE137238 data, left) and normal ovarian tissues (GSE66957 data, right). (E) Western blotting of the expressions of SOX4, endothelin‐1, and p‐AKT in HUVECs transfected with two siRNAs of SOX4 (si‐SOX4‐1, si‐SOX4‐2). (F) Representative images of tube formation and migration of HUVECs treated with si‐SOX4‐1 or si‐SOX4‐2 (scale bar = 100 μm). Total master segments length, number of master junctions, number of master nodes, and migration cell numbers were regarded as indicators of angiogenic ability in vitro and assessed by ImageJ (mean ± SD, *n* = 3). (G) Western blotting of the expressions of SOX4, endothelin‐1, and p‐AKT in HUVECs transfected with SOX4‐overexpression plasmids. (H) Representative images of tube formation and migration of HUVECs treated with SOX4‐overexpression plasmids (scale bar = 100 μm). Total master segments length, number of master junctions, number of master nodes, and migration cell numbers were regarded as indicators of angiogenesis ability in vitro and assessed by ImageJ (mean ± SD, *n* = 3). (I) Representative images of tube formation and migration of HUVECs treated with miR‐92b‐3p mimics or miR‐92b‐3p mimics plus SOX4‐plasmids. Total master segments length, number of master junctions, master nodes, and migration cells were regarded as indicators of angiogenic ability in vitro and assessed by ImageJ (mean ± SD, *n* = 3) (scale bar = 100 μm). (J) Western blotting of SOX4, endothelin‐1, and p‐AKT proteins in HUVECs cotransfected with miR‐92b‐3p mimics plus SOX4 plasmids. Data are shown by at least three independent experiments, and the Student's *t*‐test was used to compare differences. *p < .05, **p < .01, ***p < .001, ****p < .0001

miRTarbase, miRDB, miRWalk, and Targetscan were used to predict miR‐92b‐3p's potential downstream targets (Figure S2A). Among the potential targets, all databases predicted SOX4 as a candidate target with the highest score. SOX4 has been reported to enhance the promoter activity of endothelin‐1 and promote PI3K/Akt signaling. QRT‐PCR analysis illustrated the significantly decreased expression of SOX4 and endothelin‐1 mRNA, and Western blotting assay showed significantly decreased expression of SOX4, endothelin‐1, and phosphorylated AKT protein in HUVECs with miR‐92b‐3p overexpression, vice versa (Figure 4A). To verify SOX4 to be a direct target of miR‐92b‐3p, plasmids of 3′‐UTR containing a matching site or mut site were subsequently constructed (Figure S2B). Notably, dual‐luciferase assays revealed that the miR‐92b‐3p overexpression significantly inhibited reporter activity of the wt SOX4 3′‐UTR, which was totally eliminated using the mut SOX4 3′‐UTR (Figure 4B). In addition, HUVECs cultured with SKOV3‐92b/exo showed lower expression of SOX4, endothelin‐1, and p‐AKT protein than those with SKOV3/exo (Figure 4C). Because miR‐92b‐3p expression was lower in the ovarian cancer samples, we also summarized the SOX4 expression in ovarian cancer samples. The results imply that in primary ovarian cancer, SOX4 was higher than matched normal fallopian tube (GSE137238) or normal ovarian tissues (GSE66957) (Figure 4D). To further verify the effect of SOX4 on angiogenesis and migration of HUVECs, we used a SOX4 overexpression plasmid and two different small interfering RNAs of SOX4 (si‐SOX‐1 and si‐SOX4‐2) (Figure S3), and found that SOX4 inhibition effectively suppressed the expression of endothelin‐1 and phosphorylated AKT protein, and tube formation and migration of HUVECs (Figure 4E,F), vice versa (Figure 4G,H). Additionally, after cotransfecting NC‐plasmid plus NC mimics, NC‐plasmid plus miR‐92b‐3p, and SOX4‐plasmid plus miR‐92b‐3p, respectively, into HUVECs, restoring SOX4 expression partially reversed the tube formation, cellular migration and the expression of endothelin‐1 and phosphorylated AKT proteins (Figure 4I,J) suppressed by miR‐92b‐3p overexpression, suggesting that SOX4 is a target of exosomal miR‐92b‐3p and participates in exosomal miR‐92b‐3p modulating angiogenesis.

### 3.5 Exosomal miR‐92b‐3p and Apatinib represent synergistic anti‐angiogenic effect

**FIGURE 5:**
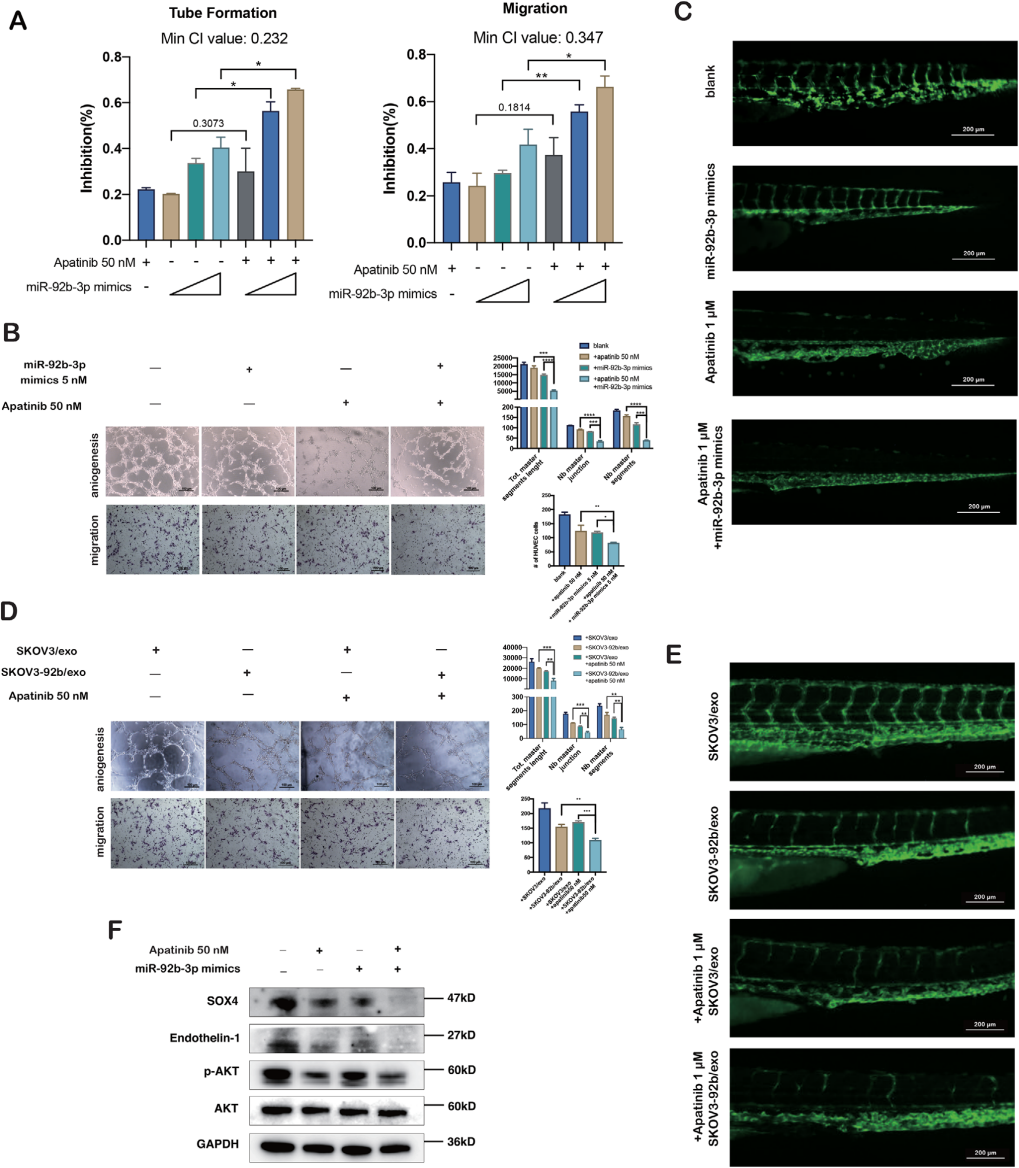
Exosomal miR‐92b‐3p and Apatinib represent the synergistic anti‐angiogenic effect. (A) Inhibition rates of tube formation and migration of HUVECs treated with Apatinib or miR‐92b‐3p mimics alone or both combined (mean ± SD, *n* = 3). In the Apatinib‐treated group, the working concentration was 50 nM. In the miR‐92b‐3p mimics group, the working concentrations were 1, 2, and 5 nM. In the combined Apatinib and miR‐92b‐3p mimics treated group, the working concentrations were 50 nM for Apatinib and 1, 2, and 5 nM for miR‐92b‐3p, respectively. Minimum CI values of tube formation and migration are shown. Data are shown as mean ± SD of the percentage of change when compared to full medium‐treated group (*n* = 3). The miR‐92b‐3p mimics treated groups were used as negative controls. (B) Representative images of tube formation and migration of HUVECs treated with Apatinib (50 nM) and miR‐92b‐3p (5 nM) (scale bar = 100 μm). Total master segments length, number of master junctions, master nodes, and migration cells were regarded as indicators of angiogenic ability in vitro and assessed by ImageJ (mean ± SD, *n* = 3). (C) Representative images of zebrafish embryos treated with Apatinib (1 μM), miR‐92b‐3p mimics, and Apatinib (1 μM) plus miR‐92b‐3p, respectively (scale bar = 100 μm). (D) Representative images of tube formation and migration of HUVECs treated with SKOV3/exo, SKOV3‐92b/exo, Apatinib, and SKOV3‐92b/exo plus Apatinib (mean ± SD, *n* = 3), respectively (scale bar = 100 μm). Total master segments length, number of master junctions, master nodes, and migration cells were regarded as indicators of angiogenic ability in vitro and assessed by ImageJ (mean ± SD, *n* = 3). (E) Representative images of zebrafish embryos treated with SKOV3/exo, SKOV3‐92b/exo, Apatinib, and SKOV3‐92b/exo plus Apatinib, respectively (scale bar = 200 μm). (F) Western blotting of the expression levels of SOX4, endothelin‐1, and p‐AKT treated by miR‐92b‐3p (5 nM) plus Apatinib (50 nM). Data are shown by at least three independent experiments, and the Student's *t*‐test was used to compare differences. *p < .05, **p < .01, ***p < .001, ****p < .0001

Apatinib suppresses angiogenesis of HUVECs by targeting VEGFR2. We tested the synergistic inhibition of combined miR‐92b‐3p and Apatinib in tumor‐associated angiogenesis. The inhibition curve and median‐effect plots of Apatinib and miR‐92b‐3p mimics were, respectively, calculated for analyzing the relationship of drug‐to‐drug interaction (Figure S4) and the combination index (CI) represented the strength of drug‐to‐drug interaction. HUVECs were treated with Apatinib (50 nM) and miR‐92b‐3p mimics (1, 2, and 5 nM), and the results showed that the smallest CI values of tube formation and migration assay were 0.232 and 0.347, respectively, suggesting that there was a strong synergism of Apatinib (50 nM) and miR‐92b‐3p mimics (5 nM) (Figure 5A,B). In addition, zebrafish embryos treated with Apatinib (1 μM) plus miR‐92b‐3p mimics for 48 h demonstrated remarkably stronger angiogenic inhibition compared to those treated with Apatinib or miR‐92b‐3p mimics alone (Figure 5C).

HUVECs were further cotreated with SKOV3/exo or SKOV3‐92b/exo and Apatinb (50 nM). The results showed that angiogenesis and migration of HUVECs treated with combined SKOV3‐92b/exo and Apatinb (50 nM) were significantly more inhibited compared with those with SKOV3‐92b/exo and Apatinb alone (Figure 5D). Similarly, the defection of newly formed intersegmental vessels in zebrafish embryos was more obvious in fish treated with combined SKOV3‐92b/exo and Apatinb than those treated with SKOV3‐92b/exo or Apatinb alone (Figure 5E). Additionally, Western blotting assay showed remarkably decreased expression of SOX4, endothelin‐1, phosphorylated AKT protein in HUVECs with Apatinib plus miR‐92b‐3p mimics compared to those with Apatinib or miR‐92b‐3p mimics alone (Figure 5F). The results together suggest that exosomal miR‐92b‐3p and Apatinib represent a synergic effect of anti‐angiogenesis.

### 3.6 Engineered RGD‐SKOV3‐92b/exo alone or combined with Apatinib inhibits tumor growth and angiogenesis in vivo

**FIGURE 6:**
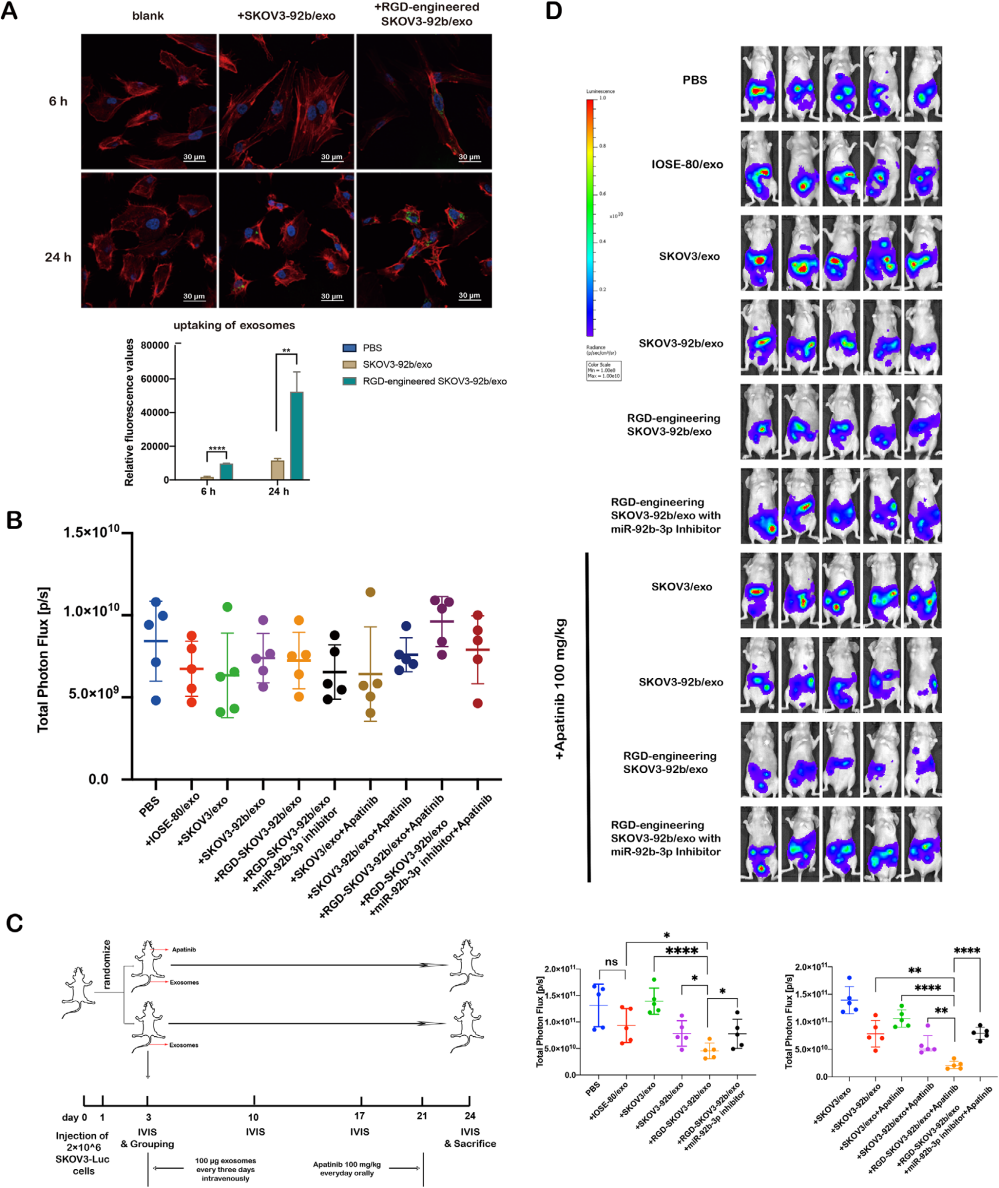
Engineered RGD‐SKOV3‐92b/exo alone or combined with Apatinib inhibits tumor growth in vivo. (A) Representative confocal microscopy images of HUVECs treated with SKOV‐92b/exo or RGD‐SKOV3‐92b/exo. F‐actin (red), nucleus (blue), and PKH67‐labeled exosomes (green) were stained (scale bar = 30 μm). Relative uptake was calculated using ImageJ software. (B) Total fluorescence values of nude mice in different groups. There were no differences among randomization groups for any of the characteristics. (C) Schematic protocol of intraperitoneal xenografted tumors in animal experiments. (D) Bioluminescence images of nude mice with different exosomal injections or Apatinib treatment. Total fluorescence values were assessed by fluorescence imaging (*n* = 5 per group). Data are shown by at least three independent experiments. The Student's *t*‐test and nonparametric test were used to compare differences. *p < .05, **p < .01, ***p < .001, ****p < .0001

**FIGURE 7:**
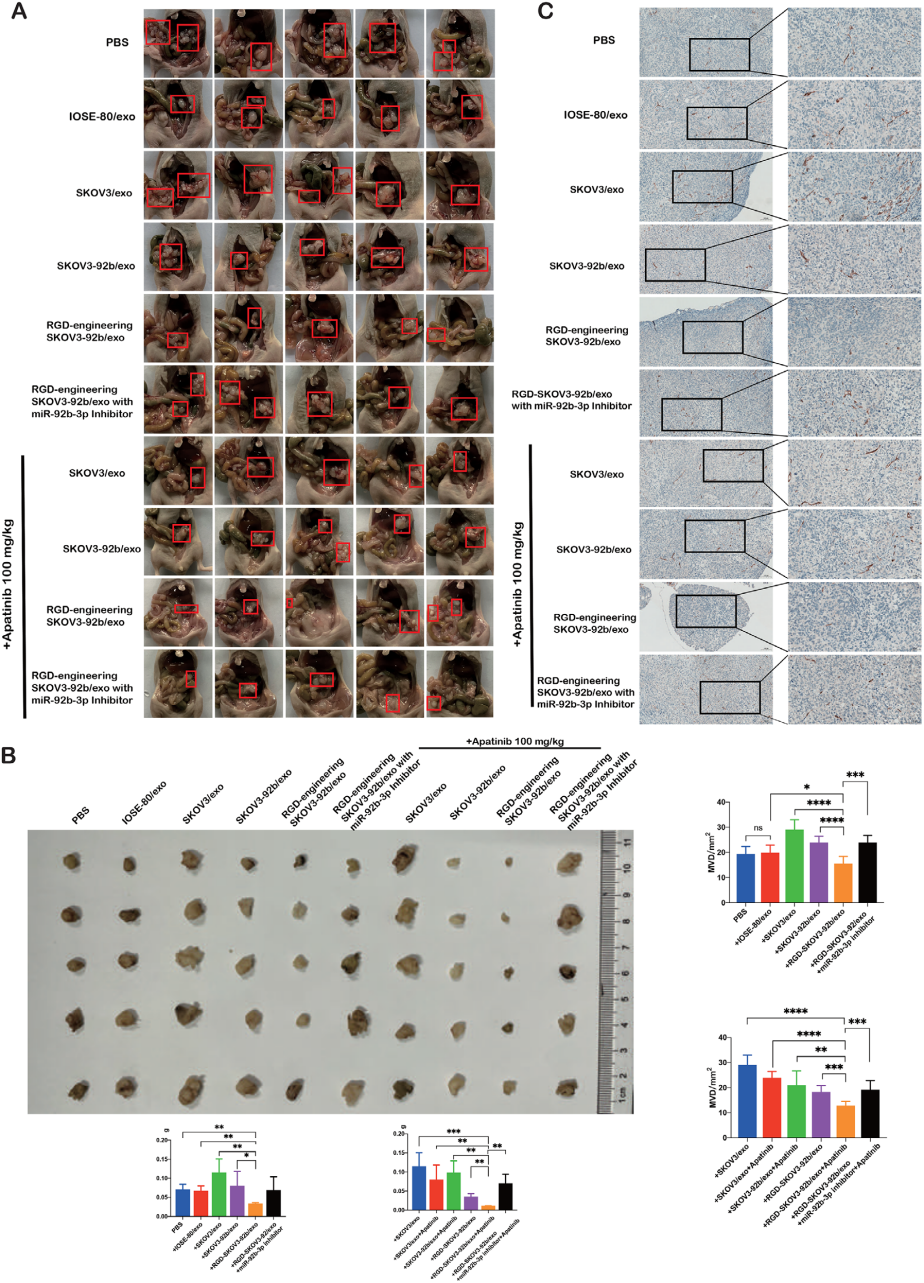
Tumor volume and angiogenesis were inhibited by engineered RGD‐SKOV3‐92b/exo alone or combined with Apatinib. (A) Gross anatomy images of nude mice in the peritoneal cavity. (B) Schematic representation of intraperitoneal xenografted tumors in different groups. Tumor size from each group was measured and weighed as represented. (C) Immunohistochemical staining of CD31 in intraperitoneal xenografted tumors (scale bar = 100 μm and 50 μm). Micro‐vessels density (MVD) was assessed by counting CD31‐positive cells (*n* = 5). Data are shown by at least three independent experiments. The Student's *t*‐test and nonparametric test were used to compare differences. *p < .05, **p < .01, ***p < .001, ****p < .0001

To estimate the potential value of exosomes carrying overexpressed miR‐92b‐3p as anti‐angiogenic agent in cancer therapy, we generated Arg‐Gly‐Asp peptide‐engineered exosomes (RGD‐SKOV3‐92b/exo) from RGD‐labeled SKOV3‐92b cells. Confocal microscopy was used to detect the amounts of engineered exosomes, and the results showed that the captured amount of RGD‐SKOV3‐92b/exo was more than that of SKOV3‐92b/exo in HUVECs (Figure S5 and Figure 6A), suggesting that RGD‐SKOV3‐92b/exo possesses stronger penetrating power into cells than parental SKOV3‐92b/exo. To further evaluate whether exosomes carrying overexpressed miR‐92b‐3p affect the viability of ovarian epithelial cells or cancer cells, CCK‐8 analysis was applied. The results showed no significant difference of cell viabilities among IOSE‐80, SKOV3, and A2780 cells treated with PBS, SKOV3/exo, SKOV3‐92b/exo, and RGD‐SKOV3‐92b/exo for 48 h, respectively (Figure S6). Then, 2 × 10^6^ SKOV3 cells with stably transfected luciferase‐plasmid (SKOV3‐Luc cells) were intraperitoneally injected into nude mice (*n* = 50). After 3 days of tumor formation, mice were randomly divided into 10 groups (*n* = 5 each group), and in vivo imaging showed the same fluorescence values among the 10 groups at that moment (Figure 6B). Mice were treated according to the schematic diagram (Figure 6C). The vivo fluorescence imaging on the 24th day showed that fluorescence amount was significantly decreased in SKOV3‐92b/exo or RGD‐SKOV3‐92b/exo groups compared to SKOV3/exo, IOSE‐80/exo, or PBS groups. Moreover, fluorescence in SKOV3‐92b/exo or RGD‐SKOV3‐92b/exo plus Apatinib groups further significantly decreased than that in SKOV3‐92b/exo or RGD‐SKOV3‐92b/exo alone group (Figure 6D). Mice were sacrificed under anesthesia on the 24th day of tumor formation. The anatomic images of abdominal cavity and tumors showed that tumor growth and metastasis were remarkably inhibited in SKOV3‐92b/exo or RGD‐SKOV3‐92b/exo with or without Apatinib (Figure 7A,B). CD31 staining was used to detect MVD, and the results showed that the changes of MVD were similar to the changes of fluorescence amount (Figure 7C). Meanwhile, there were no significant changes of body weights and organ coefficients among mice with different treatments (Figure S7A,B). HE staining images of organs are given in Figure S7C. All results together suggest that engineered RGD‐SKOV3‐92b/exo alone or combined with Apatinib inhibits tumor growth via anti‐angiogenesis in vivo.

## 4 DISCUSSION

Angiogenesis is an important feature of solid tumors.^29^, ^30^, ^31^ Tumor cells utilize neovascularization to acquire the energy for growth and metastasis. Multiple mechanisms contribute to tumor angiogenesis, in which cancer cell‐derived exosomes are also involved.^32^, ^33^ In this study, we found that exosomes from ovarian cancer cells promoted tube formation and migration of vascular endothelial cells in vitro and induced abnormal angiogenesis in zebrafish models, affirming that ovarian cancer exosomes possess the capacity to promote tumor‐associated angiogenesis.

Exosomes work through the various cargos they carry, among which miRNA is a major mediator. We performed miRNA comprehensive profiling and validated the significantly less miR‐92b‐3p amounts in exosomes form ovarian cancer cells. When HUVECs were cotreated by exosomes with less miR‐92b‐3p and mRNA transcription inhibitor DRB, miR‐92b‐3p level in HUVECs did not remarkably change, suggesting that exosomes’ stimulation does not affect internal miR‐92b‐3p expression in HUVECs. After further upregulating miR‐92b‐3p expressions of vascular endothelial cells and ovarian cancer cells, it was found that vascular endothelial cells, whether with miR‐92b‐3p overexpression or treated by exosomes from miR‐92b‐3p overexpressed ovarian cancer cells, presented suppressed angiogenesis in vitro and in zebrafish models, and vice versa. Our results suggest that exosomal miR‐92b‐3p acts as a mediator in exosomes modulating tumor‐associated angiogenesis in ovarian cancer.

miRNA functions via targeting downstream genes.^34^ We utilized mRNA target‐predicting algorithms and found that SOX4 was a candidate target of miR‐92b‐3p. SOX4 is a transcription factor as the member of the Sry‐related high‐mobility group box (SOX) family, and has been recognized to promote the development of various cancers.^35^, ^36^, ^37^ A recent study showed that SOX4 also played a role in angiogenesis of breast cancer.^38^ In our study, it was found that SOX4 was directly bound to miR‐92b‐3p by gene function experiments, revealed the opposite effect as miR‐92b‐3p on angiogenesis, and further, upregulated SOX4 partially reversed the decreased angiogenesis induced by upregulated miR‐92b‐3p, suggesting that exosomal miR‐92b‐3p modulates angiogenesis via targeting SOX4 in ovarian cancer cells.

Along with the progress of the technology, exosome used as drug delivery vehicle is becoming a new approach to treat various diseases. As a vesicle with lipid membrane, exosome provides good protection for cargos from blood flow degradation. And as a vehicle much smaller (30–150 nm) than other drug transportations, such as EnGeneIC Delivery Vehicle (EDV) nanocells (400 nm),^39^ and the homology of cell membrane, exosome is easier to enter the cell and play a role.^40^ There are various sources of exosomes as drug carriers, including mesenchymal stem cells (MSCs),^41^ dendritic cells (DCs),^42^ 293T cells,^43^ and cancer cells.^44^ However, there are doubts about the potential risk using cancer cells as donor cells of exosomes in potential clinical applications. Theoretically, it is possible that exosomes derived from cancer cells carrying unknown cargos promoting the recurrence or metastasis, but it is needed to obtain evidence to support this hypothesis. Some studies reveal that RGD peptide contains Arg‐Gly‐Asp sequence that can specifically bind to the α_v_β_3_ integrin, which is highly expressed on the surface of blood vessel endothelial cells.^45^, ^46^ Therefore, RGD‐engineered exosomes possess the ability to target vessel epithelial cells rather than cancer cells. Thus, we artificially generated miR‐92b‐3p overexpressed engineered exosomes that are conjugated with a peptide containing the Arg‐Gly‐Asp (RGD) sequence (RGD‐SKOV3‐92b/exo) to enhance the ability of miR‐92b‐3p exosomes of targeting the blood vessels. As expected, we found that RGD‐SKOV3‐92b/exo significantly strongly inhibited transplanted tumor growth and angiogenesis than SKOV3‐92b/exo in nude mouse models, suggesting that engineered exosome with overexpressed miR‐92b‐3p may have the potential to be used as an anti‐angiogenic agent.

We further investigated the anti‐tumor effect of SKOV3‐92b/exo or RGD‐SKOV3‐92b/exo combined with Apatinib. Apatinib is a small‐molecule TKI that selectively binds to and potently inhibits VEGFR‐2 activity and then suppresses angiogenesis.^47^ The anti‐angiogenic effect of Apatinib has been revealed in a variety of tumors,^48^ but is not as good in ovarian cancer as in other cancers. Interestingly, we confirmed a strong synergetic inhibition of SKOV3‐92b/exo and Apatinib on angiogenesis in vitro. Compared to SKOV3‐92b/exo, we also observed stronger inhibitory effect of RGD‐SKOV3‐92b/exo whether alone or combined with Apatinib on tumor growth and angiogenesis in nude mouse models. Although the involved mechanism still needs to be further investigated, our results may provide a new approach for combined anti‐angiogenic therapy of ovarian cancer.

In summary, our study showed reduced miR‐92b‐3p amount in exosomes form ovarian cancer cells, exosomal miR‐92b‐3p inhibits tumor growth and tumor‐related angiogenesis via targeting SOX4, and artificially generated exosomes with overexpressed miR‐92b‐3p (RGD‐SKOV3‐92b/exo), whether alone or combined with Apatinib, exert a strong inhibition of tumor growth via anti‐angiogenesis in vivo. Our findings demonstrate the effect and mechanism of exosomal miR‐92b‐3p from ovarian cancer on tumor‐associated angiogenesis and the potential of artificially generated exosome with overexpressed miR‐92b‐3p to be used as anti‐angiogenic agent, which may provide a new approach for anti‐angiogenic therapy of ovarian cancer.

## 5 CONCLUSIONS

Our experiments demonstrate that compared to the exosomes from immortalized ovarian surface epithelia cells, exosomes from ovarian cancer cells with downregulated miR‐92b‐3p promote the tumor‐related angiogenesis by influencing the angiogenic and migration abilities of vascular endothelial cells. Experiments suggest that the enrichment of exosomal miR‐92b‐3p has strong suppressing effects on tumor‐related angiogenesis and tumor growth of ovarian cancer, whether alone or with Apatinib. We also artificially generated a novel kind of exosomes with overexpressed miR‐92b‐3p and superior targeting of vascular endothelial cells by conjugating with a peptide. The new kind of exosomes exhibits a stronger inhibition of tumor‐related angiogenesis and tumor growth in ovarian cancer, whether alone or with Apatinib. Our findings provide evidence of the potential antitumor effect of engineered exosomes with overexpressed miR‐92b‐3p in ovarian cancer, and suggesting a new approach of combined anti‐angiogenic therapy with Apatinib in first diagnosis of ovarian cancer.

## CONFLICT OF INTEREST

The authors declare that there is no conflict of interest.

## AUTHOR CONTRIBUTIONS

Xing Xie and Xiaodong Cheng created the study concept. Jiaying Wang, Conghui Wang, and Yang Li designed the study and drafted the manuscript. Jiaying Wang, Conghui Wang, Yang Li, Mingyue Li, Tingjia Zhu, and Zhangjin Shen contributed to data acquisition and/or analysis. Hui Wang, Weiguo Lv, and Xinyu Wang provided technical or material support. Jiaying Wang and Conghui Wang performed statistical analyses. Xing Xie, Xiaodong Cheng, and Yang Li directed and supervised the study.

## Supporting information

Supporting Information

## Data Availability

The data that support the findings of this study are available from the corresponding author upon reasonable request.
