# Supplementary material for: Potential of peptide‐engineered exosomes with overexpressed miR‐92b‐3p in anti‐angiogenic therapy of ovarian cancer
Source: Clin Transl Med. 2021 May 18;11(5):e425. doi: 10.1002/ctm2.425 (PMC8131502; doi:10.1002/ctm2.425)
Supplement: Supplementary file 1 — Supporting Information [file CTM2-11-e425-s001.pdf]

Supplementary Materials:

Fig.S1

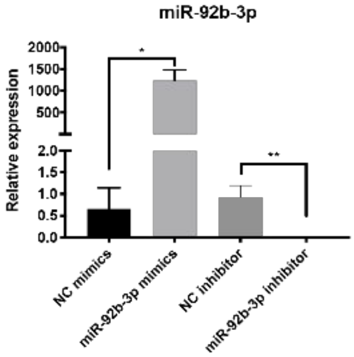

Supplementary Figure 1. Transfection efficiency of miR-92b-3p mimics or inhibitor.

The qRT-PCR analysis on the transfection efficiencies of miR-92b-3p mimics and inhibitor (mean ± SD, n = 3).

Data are shown by at least three independent experiments and the Student t-test was used to compare differences. *P < 0.05, **P < 0.01.

Fig.S2

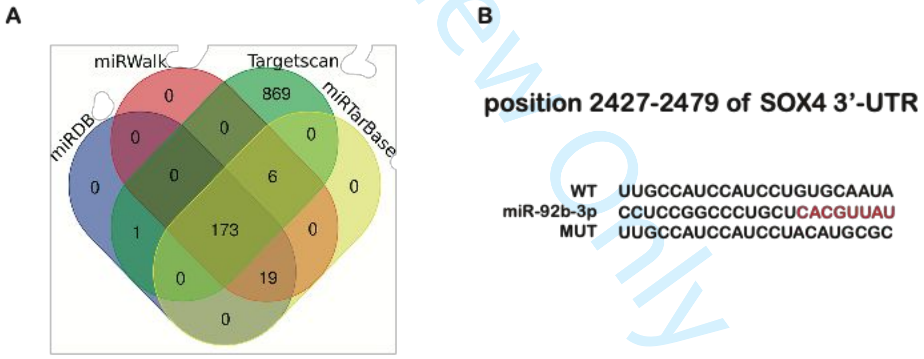

Supplementary Figure 2. Prediction of target genes of miR-92b-3p and binding site with SOX4.

(A) Venn diagram of the total numbers of common targets among four prediction databases (miRDB, miRWalk, Targetscan and miRTarBase).

(B) Schematic representation of pmirGLO-derived reporters bearing the binding site of SOX4 3′UTR or the 3′UTR mutating sequences.

For Review Only

Fig.S3

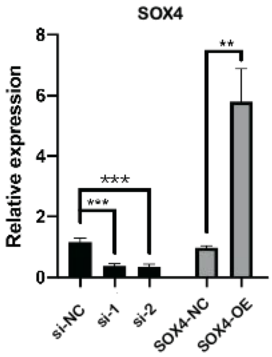

Supplementary Figure 3. Transfection efficiency of SOX4-siRNAs and SOX4-plasmids.

The qRT-PCR analysis on the transfection efficiencies of SOX4 siRNAs and plamids (mean ± SD, n = 3).

Data are shown by at least three independent experiments and the Student t-test was used to compare differences. **P < 0.01, P***<0.001.

For Review Only

Fig.S4

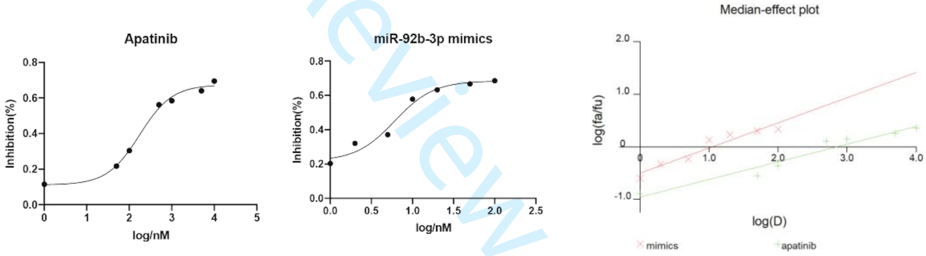

Supplementary Figure 4. Inhibition curve of miR-92b-3p and Apatinib on angiogenesis in vitro.

The inhibition curves and median-effect plots of miR-92b-3p mimics and Apatinib.

Fig. S5

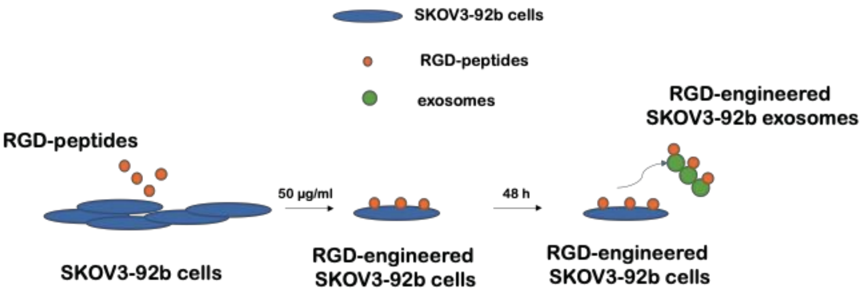

Supplementary Figure 5. Schematic diagram of the new type of exosomes construction.

Schematic illustration for realization of RGD-engineered SKOV3-92b/exo. RGD-SKOV3-92b/exo was extracted from supernatant of SKOV3-92b-3p cells incubated with conditional medium with 50 μg/ml RGD for 48 h.

Fig.S6

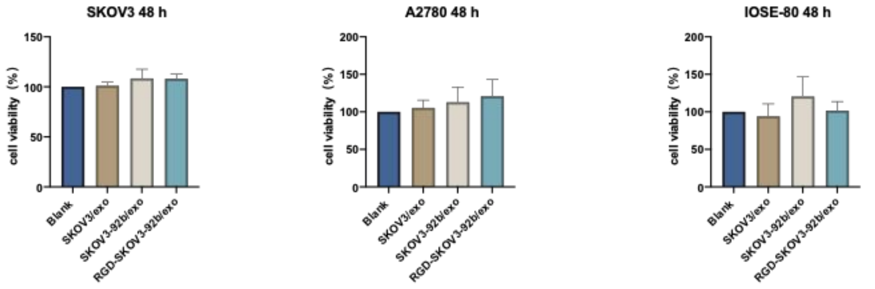

Supplementary Figure 6. Cell viability of IOSE-80, SKOV3 and A2780 cells treated with PBS, SKOV3/exo, SKOV3-92b/exo and RGD-SKOV3-92b/exo for 48 h.

Cell viability assay verified that the there were no differences between the groups treated with SKOV3-92b/exo or RGD-SKOV3-92b/exo, compared to those groups treated with SKOV3/exo.

Fig.S7

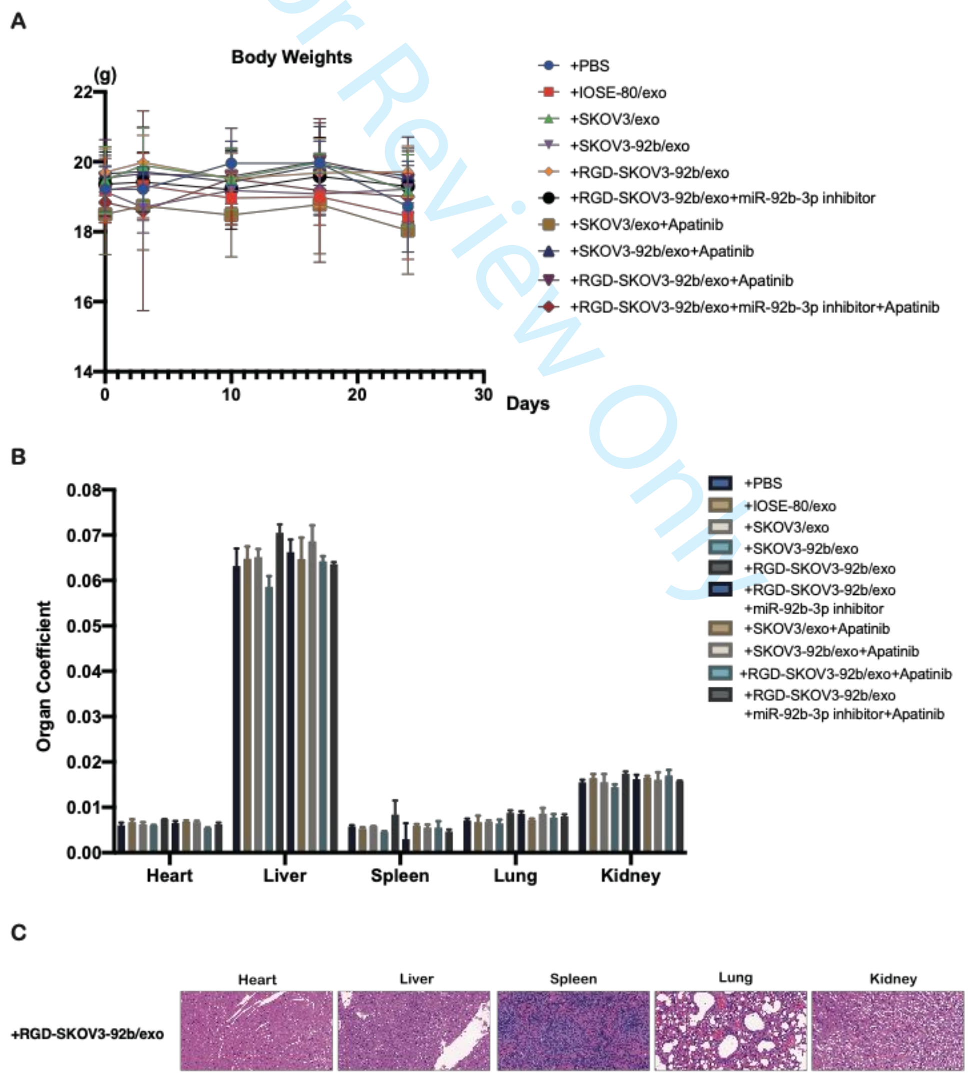

Supplementary Figure 7. Physiological state of nude mice

For Review Only

(A) Weight changes in nude mice.
(B) Organs coefficients in nude mice.
(C) Representative HE staining images of organs in nude mice.

For Review Only

Table S1. The primer sequences

**RNAs**	**Primer (5'-3')**
miR-92b-3p	Reverse Transcription Primer<br>CTCAACTGGTGTCGTGGAGTCGGCAATTCAGTTGAGGGAGGCC<br>Forward Primer<br>ACACTCCAGCTGGGTATTGCACTCGTCCCGGC<br>Reverse Primer<br>TGGTGTCGTGGAGTCG
U6	Reverse Transcription Primer<br>AACGCTTCACGAATTTGCGT<br>Forward Primer<br>CTCGCTTCGGCAGCACA<br>Reverse Primer<br>AACGCTTCACGAATTTGCGT
GAPDH	Forward Primer<br>GGAGCGAGATCCCTCCAAAAT<br>Reverse Primer<br>GGCTGTTGTCATACTTCTCATGG
SOX4	Forward Primer<br>ACCGCACGCCAAGCTCATCC<br>Reverse Primer<br>GTCCGCGCCTTGTACAGCGA
Endothelin-1	Forward Primer<br>TTGAGATCTGAGGAACCCGC<br>Reverse Primer<br>GCTCAGCGCCTAAGACTGTT

For Review Only

Table S2. Antibody names, companies and dilution ratios

**Antibodies**	**Company and Dilution**
CD63	EXOAB-CD63A-1, SBI (1: 1000)
Hsp70	EXOAB-Hsp70A-1, SBI (1: 1000)
GAPDH	db106, Diagbio (1: 2000)
SOX4	A10717, Abclonal (1: 1000)
Endothelin-1	A0686, Abclonal (1: 1000)
p-Akt	4060, Cell Signaling Technology (1: 2000)
Akt	4685, Cell Signaling Technology (1: 1000)

For Review Only

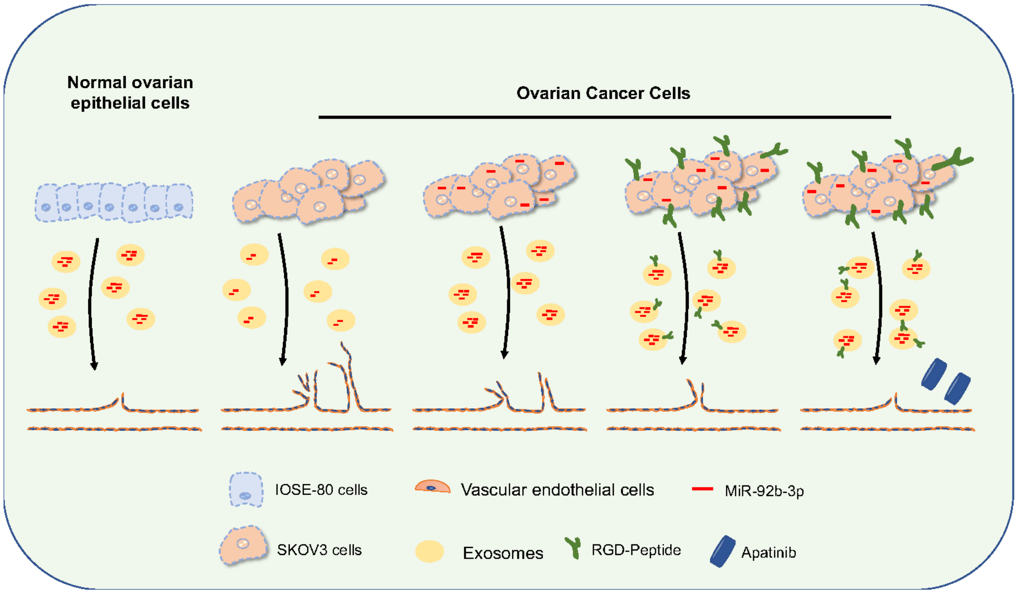

Headlights：

1. MiR-92b-3p is significant lower in exosomes derived from ovarian cancer cells than that derived from normal ovarian epithelial cells.

2. Exosomes with overexpressed miR-92b-3p can inhibit the angiogenesis of ovarian cancer.

3. DSPE-PEG2K-RGD modified engineered exosomes loaded with miR-92b-3p can produce a combined anti-tumor and anti-angiogenetic effect with Apatinib in nude mice abdominal tumor models.
